# You are the Key to Your Success!

**DOI:** 10.4103/0972-0707.55616

**Published:** 2009

**Authors:** D Kandaswamy

**Affiliations:** Department of Conservative Dentistry and Endodontics, Sri Ramachandra Dental College and Hospitals, Chennai, India

Hi folks,

We have all seen Amir Khan's recent hit film *Ghajini*. Amir Khan was willing to shave his head for the film. Do you think Amir Khan would have sacrificed both his hands for Rs. 20 crore! Leave alone the super star, do you think a beggar on the streets would sacrifice his eyes or even legs for Rs 20 crore! You know the clear answer. All the working components of our body are very precious but we don't realize its value until we are asked to part with it. The *human body* is God's greatest gift to humanity/mankind. I am sure you will agree.

Imagine a situation in which God says: “Keep pumping your heart voluntarily or it will stop”. Even if a beautiful lady comes to kiss, we would probably say, please lets get done with this fast lest I should die if I stop pumping my heart”.

A fully automated working human body is the most wonderful gift anybody could ever receive. It helps us do good or bad, feel happiness or sadness, be magnanimous or vengeful. We can either enjoy all our blessings or complain about our bliss. The choice is * purely ours* . The best choice would be to be happy, joyful, magnanimous, helpful and nice to yourself and others. We all know about Mahatma Gandhi's non-violence. All of us infer that non-violence means not harming anybody. But little do we realize that non-violence towards one's own self is equally important. And so, please remove all those feelings which can act against your body - anger, envy or sorrow. Prepare for a happy future by giving yourself the right present.

When I was in Government service I used to tell my students and staff - “Accept your limitations and excel within limitations”. When I shifted to the private sector, I realized that this statement holds true there too; contrary to my belief that it is valid only within Government settings. And suddenly it dawned upon me that this statement holds true in one's own life as well.

My only friendly advice to the young Post-Graduates (PG) in this faculty is: “You may or may not be blessed with good faculty, good department, good equipment, good library or even a good guide. But you have two *choices* : i) brood, complain and feel sad about your fate or (ii) find a way within your limitations to excel. You have walked into this specialty as a ‘*nobody*’, but choose to leave the department as a ‘*somebody*’. Look for opportunities to excel, be distinct and different at your seminars, journal clubs, library dissertation, thesis, everyday case presentations or even routine clinical work. Make sure your class shows in everything you do.”

Today, there are a lot of young heads of departments (HODs) in the country. As a senior HOD (even though I hate to admit I am old) I take liberty to give a little friendly advice. You may or may not be blessed with good PGs, good equipment, good supporting staff or good academic material. Again, look for opportunities in everything you do, be it a theory class, presiding over a seminar or guiding a thesis. Do not differentiate among your PGs. Treat them equally and give them a lot of importance. A good teacher is known by his students' performances. Strive to be that teacher whom every aspiring somebody idolizes and wants to be a disciple of; that teacher who stands out in a group.

I take this opportunity to thank the editor of this journal, a wonderful youngster, for giving me this opportunity to share my thoughts. 

“Count your blessings, they are in plenty and be that somebody who matters”.

Long live FODI.

